# The Book World of Medicine and Science

**Published:** 1911-08-05

**Authors:** 


### The Book World of Medicine and Science.

Fever-Nursing. By J. C. Wilson, M.D., A.M. Sixth Edition, revised and enlarged. (Philadelphia and London: J. B. Lippincott Co. 1910. Pp. 259. Price 5s.)

While following the plan on which this text-book of practical nursing was originally written, the author has endeavoured to eliminate all obsolete matter and unpractical detail. The sixth edition, which has recently been published simultaneously in the United States and in this country, seems to us to be on the whole a very satisfactory manual on fever nursing. We can recommend it to the attention of English nurses, for the excellence of its teaching of general principles makes amends for the occasional defects due to its American origin. The first three chapters, comprising nearly half the book, deal with a variety of subjects connected with fever, hygiene, and nursing in general. The sphere of the nurse is fairly well delimited from that of the physician, but a more or less successful attempt is made to explain the why and the wherefore of possible methods of treatment. It is in the matter of the details of treatment that one finds opportunity to cavil here and there, but we concede that, from the nursing point of view the author has compiled three most useful chapters. We fail, however, to see the necessity of mentioning temperatures of 150° and 170°, especially as the whole paragraph leaves the impression that the author is not quite convinced of their impossibility. The subject of temperature charts and pyrexia is fully explained, but the observations on clinical thermometers is in need of revision.

In the second half of the book the individual fevers are considered. The chapter on the continued fevers contains a good account of enteric fever with a full description and earnest advocacy of the cold-bath treatment. Compared with the short accounts of some of the eruptive fevers, the space devoted to typhus must seem to English readers unduly long; but this section, including also yellow fever and dengue, is interestingly written, and is of course an important one for American nurses. Some very excellent plates give exceptionally good views of certain aspects and eruptions of a few of the diseases, particularly of smallpox; but the chapter on the eruptive fevers would be improved by extension and amplification here and there: for example, there is not sufficient stress laid on the importance and the frequency of broncho-pneumonia as a complication of measles. Another point which it is somewhat surprising to find but briefly mentioned is the life-history of the malarial parasite, which surely deserves a more extended notice from the point of view of the prophylactic treatment of malaria. In the concluding chapter, pneumonia and rheumatic fever are included with diphtheria and cerebro-spinal fever. The accounts are usefully compressed but sufficiently explicit, though they also stand in need of some alteration to give them a more modern ring.

This text-book does indeed make profitable reading for nurses, but we cannot proclaim it superior to the best of our home-made nursing manuals; considering its small compass and moderate price, it should continue to enjoy the favour which six editions seem to imply it has already met with.

Cholera and Its Treatment. By Leonard Rogers, M.D., F.R.C.P., F.R.C.S., I.M.S. (London: Hodder and Stoughton and Henry Frowde. 1911. Pp. 236. Price 10s. 6d. net.)

A severe test of the successful composition of a treatise on a special subject is its power of sustaining the attention of readers who may be considered only remotely interested in the subject dealt with. Though it cannot be said, particularly at the present time, that the treatment of cholera is without practical interest to the general practitioners of this country, it must be conceded that, fortunately, it is a subject outside everyday work. Yet we venture to urge all medical practitioners to read this very fascinating book, because it is a most delightful example of the value of physical and chemical science applied to clinical therapeutics and because it will be read with a great deal of pleasure, even by those who have never treated a case of cholera, owing to the direct and practical tone of its matter. As an antidote to therapeutic nihilism, it can be confidently recommended.

The author enables us to follow the steps in the evolution of a method of treatment which has already reduced the death rate of cholera to less than half what it had been. From observations on the specific gravity of the blood made in a manner sufficiently simple for the bedside, Dr. Rogers demonstrates the frequency with which a clear indication for transfusion is obtained in the acute stages of the disease. A specific gravity of 1,063 to 1,065 means a loss of about half the fluid from the blood, while sometimes the s.g. has been as high as 1,076. But in conjunction with this must be considered the important fact that in severe cases there is a loss of salts as well as fluids, so that the chlorides in the serum were found to be lower than normal, instead of higher in proportion to the loss of fluid, as would have been the case if no salts had disappeared from the circulation.

A series of observations on the blood pressure has led Major Rogers to regard a pressure of 70 mm. or under as an indication of the presence of a dangerous degree of collapse, calling for an intravenous saline injection immediately, even if the patient's general condition at the moment should appear to be fairly good; for a single copious evacuation might produce a fatal collapse. The logical conclusions deduced from these and other observations are applied practically in the system of treatment which has produced such excellent results. The use of massive saline infusions is, of course, not new; but with the results of his researches before him, the author has been able to avoid some of the untoward consequences and the only transient benefits formerly obtained by the employment of normal saline solutions.

To quote from the book, it is found that "by the use of hypertonic solutions on the other hand the saline content of the blood is immediately raised very considerably, with the result that the osmotic currents will tend to carry more fluid into the blood rather than to allow it to escape from it, and thus the diarrhœa is checked instead of encouraged, as by normal salt solutions." The author prefers the intravenous method of injection, the technique of which is given in detail, and the strength of the saline solution most suitable for general adoption works out at 1.4 per cent., including small quantities of the chlorides of calcium and potassium with the sodium chloride.

The clinical description of cholera, including differential diagnosis, forms an admirable essay, which contains many points of great interest. That portion of the chapter dealing with the complication of uræmia, makes reference again to the subject of the blood pressure. During the performance of his researches, Major Rogers noticed that every case in which the blood pressure remained permanently below 100 mm. died of uræmia, while no cases in which it rose to over 105 mm. succumbed to renal complications. Further experiments led to the conclusion that the continued suppression of urine during the reaction period was caused by actual mechanical obstruction to the circulation through the kidney; hence it becomes a matter of the first importance to raise the blood pressure to over 100 mm. at an early stage in the treatment of cholera.

In another direction a most useful help has been employed—namely, the administration of permanganate salts as an oxidising agent for the purpose of destroying the toxins in the alimentary canal. Major Rogers has given very large doses of permanganates without any deleterious consequences, and is strongly impressed by their satisfactory effect on the stools.

Besides the section devoted to treatment, which is naturally of the greatest interest, the volume contains an historical survey of previous epidemics. This portion of the subject is admirably compiled, and can be comfortably followed with the aid of excellent maps. The story of the slow acceptance of scientific truths and the fallibility, not to say blindness, of official reports, is an object-lesson to be taken to heart and calmly reflected upon.

We have, perhaps, indicated sufficiently the practical advances in treatment which are to be found in this very satisfactory treatise, the merits of which we feel confident will be speedily recognised.

Salads and their Cultivation. Edited by T. W. Sanders, F.L.S., F.R.H.S. Illustrated. (London: W. H. and L. Collingridge. 1911. Price 1s. net.)

Although much has been written of the cultivation of vegetables for salad purposes in books dealing with the cultivation of vegetables generally, hitherto no work has been published exclusively on the subject, and it is to remedy this defect that the present volume has been published. Those desirous of information on salads cannot do better than consult this handbook, which gives in a short space the main facts connected with the history, culture, storing crops, varieties, pests, and diseases of all the chief vegetables in common use as salads. The little book also contains a chapter on salad recipes, which should prove useful to the housewife in search of variety.

The Story of Bacteria. By T. Mitchell Prudden, M.D. Second Edition, revised and enlarged. Illustrated. (London and New York: G. P. Putnam's Sons. 1910. Price 3s. 6d.)

It is rare to come across so pleasantly written a book on popular science as this little treatise on bacteriology by Dr. Prudden. The difficulties of the subject have been successfully overcome and the story has been told in an exceptionally lucid manner. The author has, it may be conceded, the advantage of dealing with a subject fascinating in itself, but to him is due the credit of preserving the reader from tedious technicalities and yet giving an account which even medical men could read with profit. The intelligent lay reader will be able to gain a clear conception of the working of bacteria and their great importance in many processes of everyday life. Among the subjects touched upon, we find entertaining accounts of the nitrifying organisms and their importance to the farmer, of the commoner pathogenic germs, of phagocytosis, of the dangerous rôle of the house-fly and a prosaic explanation of the miracle of the Bleeding Host. The illustrations are good and easily comprehended.

